# Minute tubercles in bitterling larvae: Developmental dynamic structures to prevent premature ejection by host mussels

**DOI:** 10.1002/ece3.6321

**Published:** 2020-05-03

**Authors:** Hyeong Su Kim

**Affiliations:** ^1^ Inland Aquaculture Research National Institute of Fisheries Science Changwon Republic of Korea

**Keywords:** bitterling, coevolution, freshwater mussel, host–parasite, minute tubercle, premature ejection

## Abstract

1. Bitterlings are small freshwater fish that use long ovipositors to lay eggs in host mussels, and they have morphological adaptations to increase larval survival. The most well‐known adaptation is the minute tubercles on the skin surface of larvae; they are developed in early‐stage larvae with weak swimming ability and disappear in free‐swimming larvae before they leave the host mussel.
2. In the present study, I comprehensively analyzed the developmental stages of *Rhodeus pseudosericeus* larvae, their morphological and physiological characteristics, their migration inside mussels, and the development of minute tubercle in order to elucidate the morphological function of the minute tubercles. These tubercles began to develop 1 day after hatching (formation stage), grew for 2–5 days (growth stage), reached the peak height after 6–7 days (peak stage), abruptly reduced in height after 8–10 days (abrupt reduction stage), and gradually reduced in height (reduction stage) until completely disappearing 27 days after hatching (disappearance stage).
3. The larvae remained in the mussels’ interlamellar space of the gill demibranchs until 10 days after hatching and began to migrate to the suprabranchial cavity 11 days after hatching. At this time, the larvae had a heart rate and the caudal fin began to develop. At 24 days after hatching, the minute tubercles had almost disappeared, and some individuals were observed swimming out of the mussels.
4. The results presented herein elucidate that the minute tubercles are the developmental dynamic structures that the bitterling larvae have morphologically adapted to prevent premature ejection from the mussel.

## Graphical abstract

In the present study, my aim was to investigate whether the development of minute tubercles in bitterlings prevents premature ejection by host mussel. The Figure 4 showed the major results in the this study: position inside the mussels, development of minute tubercle on skin surface, and the morphological characteristics of bitterling larvae.

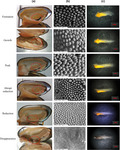

## 1 INTRODUCTION

Coevolution is a process that involves reciprocal evolutionary changes resulting from the interrelationship between a group of organisms and associated populations and plays an important role in the adaptation and speciation of almost all living organisms (Thompson, 1994). Prey–predator, host–parasite, and symbiont relationships are examples of coevolution (Liu, Zhu, Smith, & Reichard, 2006). Theoretical evidence of coevolution and adaptive traits has been obtained mainly from studies on host–parasite interactions (e.g., avian brood parasitism) (Davies, 2000; Rothstein & Robinson, 1998; Takasu, 1998; Thompson & Burdon, 1992).

Oviparous fishes with parental care utilize different reproductive strategies to select and prepare spawning sites to increase the number and survival rate of larvae, for instance, by defending their eggs or oxygenating the water around them (Smith & Wootton, 1995). Besides the reproductive success of an individual, the choice of type and site of spawning in species with parental care are important factors that influence larval survival (Kitamura, 2005; Mills & Reynolds, 2002; Refsnider & Janzen, 2010; Smith, Reynold, Sutherland, & Jurajda, 2000). In contrast, species without parental care are vulnerable to abiotic (e.g., low oxygen rates and extreme temperatures) and biological factors (e.g., predators, parasites, and competitors) during the larval stages (Smith & Wootton, 1995).

Bitterlings (Acheilognathinae) are small freshwater fish predominantly distributed in Europe and Northeast Asia, and they have a unique relationship with freshwater mussels (Bivalvia: Unionidae) (Damme, Bogutskaya, Hoffimann, & Smith, 2007; Smith, Reichard, Jurajda, & Przybylski, 2004). During the spawning season, the female bitterlings elongate their ovipositors and spawn on the gills of mussels through the mussels’ exhalant siphons. The male fish, which have nuptial coloration and form territories around the mussels, release sperms that enter the mussels’ inhalant siphons during their feeding and breathing activities. Therefore, the eggs are fertilized in the gill cavity of mussels where, depending on the temperature, they remain for 3–4 weeks feeding on their own reserves until they become free‐swimming larvae, which leave the mussels and begin external feeding (Aldridge, 1999; Smith et al., 2004).

Although bitterlings do not exhibit parental care, they lay very few eggs. Moreover, it is difficult for the bitterlings to lay their eggs inside mussels, but they safely spend their early developmental stage in the mussels (Kitamura, 2008; Smith et al., 2004; Zale & Neves, 1982). Host–parasite interaction and choice of oviposition site, critical aspects of vertebrate ecology, have not been sufficiently studied (Refsnider & Janzen, 2010). The relationship between bitterlings and mussels is a notable example of coevolution of a host and parasite (Mills & Reynolds, 2003; Reichard et al., 2010; Reynolds, Debuse, & Aldridge, 1997; Rouchet et al., 2017).

Recent studies have shown that the bitterling–mussel relationship is in fact a type of host–parasite interaction (Mills & Reynolds, 2003; Reichard, Bryja, Polacik, & Smith, 2001; Spence & Smith, 2013). To prevent ejection by host mussels, bitterlings have several unique physiological, behavioral, and morphological adaptations (Aldridge, 1997; Kitamura, 2006a; 2006c; Methling et al., 2018; Smith et al., 2004; Spence & Smith, 2013). The fish larvae develop single‐celled epidermal cells, called “tubercles,” on their skin surface, which are known to play an important role in preventing the larvae from being prematurely ejected from the gills (Suzuki, Akiyama, & Hibiya, 1985; Suzuki & Hibiya, 1984a; 1985; Suzuki & Jeon, 1987). Minute tubercles are common in all developmental stages of bitterlings, although the larval morphology is diverse. Previous studies have reported that the minute tubercles are mainly developed in the frontal part of the larvae and on the eyes and a wing‐like projection (Kim, Park, Park, Kang, & Kim, 2008; Park, Oh, Kim, Kang, & Beon, 2008; Suzuki & Jeon, 1988a; ).

Several studies using in vitro insemination have briefly described the development of the minute tubercles and the morphological characteristics of larvae, and based on their results, the minute tubercles were assumed to prevent premature ejection of larvae from their host mussels. However, no comprehensive studies correlating the developmental stages of larvae with their morphological and physiological characteristics, their migration inside the mussels, and the development of the minute tubercles have been conducted. Therefore, in the present study, my aim was to investigate whether the development of minute tubercles in bitterlings prevents premature ejection. For this, I focused on the relationships among the height of the minute tubercles, morphological and physiological characteristics of the larvae during development, and position of the larvae in the mussels. Furthermore, I discuss the evolutionary advantages of the development of the minute tubercles and migration of larvae inside mussels to increase survival.

## 2 MATERIALS AND METHODS

### 2.1 Study site

This study was performed in the Heukcheon Stream of the Namhangang River in Yangpyeong‐gun, Kyeongki‐do, Korea in March‐April 2017. The experiment site was a small pond (30 m wide × 10 m long; maximum and mean depth of 1.5 and 0.8 m, respectively) connected to the Heukcheon stream. The Stream bed is comprised of silt and mud. The site had eight species of freshwater fishes, but only one bitterling species, *Rhodeus pseudosericeus*, and one mussel species, *Unio douglasiae sinuolatus*. *R. pseudosericeus* is considered an endangered species by the Ministry of the Environment of Korea; thus, I received permission from the Ministry of Environment of Korea (Permission number, 2017‐22) to perform this study.

### 2.2 Induction of R. pseudosericeus spawning

To study the development of the minute tubercles in each larval developmental stage and the position and migration of larvae in mussels, *R. pseudosericeus* spawning on mussels was simultaneously induced in large quantities. Mussels were collected on 5 March 2017, before the spawning period of *R. pseudosericeus*. A total of 150 mussels (shell length 35–70 mm) were collected using a kick net (mesh size 3 mm × 3 mm) and placed in the small pond where the spawning experiments would be conducted. The captured mussels were then placed on fine sand inside a plastic box (600 mm length × 600 mm width × 200 mm height) through which water could pass but not *R. pseudosericeus* individuals. The sealed box was placed in another pond next to the pond where the experiment would be performed. Spawning was induced on 27 March 2017, at night. The mussels were then divided into three boxes with 50 mussels each, and the boxes were placed at 3‐m intervals. On the morning of 29 March 2017, 36 hr later, the plastic box containing the mussels was removed to complete the spawning induction experiment. Water from the pond was collected in three plastic boxes (1,000 mm length × 1,000 mm width × 600 mm height), which were transferred to a laboratory with an oxygen generator.

### 2.3 Mussel rearing in the aquarium

In the laboratory, an experimental aquarium (600 mm width × 600 mm length × 600 mm height) was prepared, and sand was evenly spread (100 mm height) at the bottom of the aquarium. To induce spawning, the three groups of 50 mussels were separately placed in three glass tanks. Oxygen was supplied so that dissolved oxygen (DO) was maintained above 7 mg/L, and aquarium heaters were used to maintain the water temperature around 20°C ± 1°C. A natural 13/11 hr light:dark photoperiod was used. The mussels were maintained in the experimental aquarium and fed daily with a suspension of live *Chlorella* sp. derived from an indoor aquarium.

### 2.4 Observation of R. pseudosericeus larval development stages and position inside the mussels

After 1 day in the tanks, three mussels per day for 30 days were checked for *R. pseudosericeus* larvae. The presence of larvae on the four gills (left or right, outer or inner) of *U. d. sinuolatus* mussels was checked using a mussel‐opening device that enabled mussels to be opened to approximately 10 mm. The adductor muscle of mussels was cut to examine the position, number, and developmental stage of bitterling eggs/embryos/larvae. Mussels without bitterling eggs/embryos/larvae were housed in different tanks.

**FIGURE 1:**
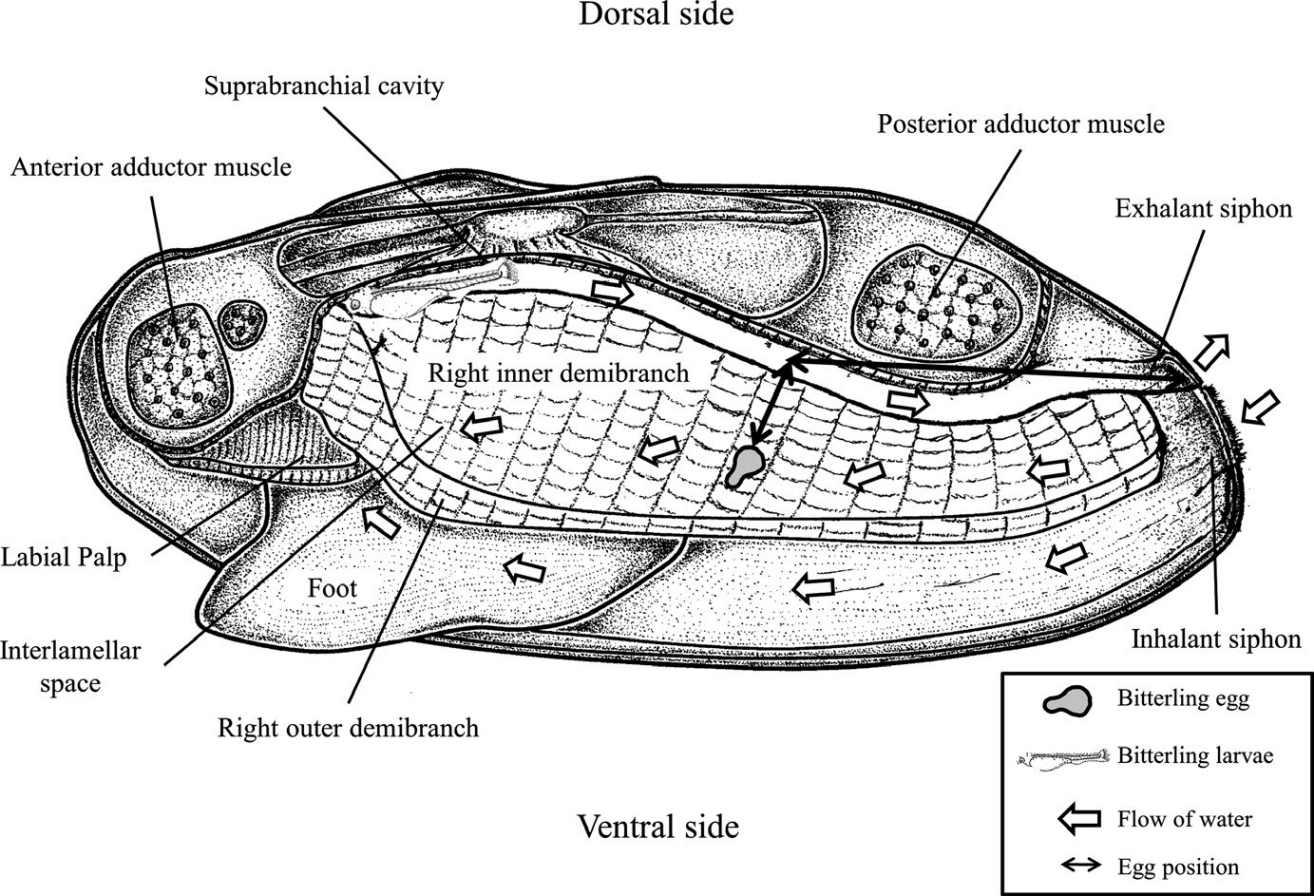
Diagrammatic anterior‐posterior cross‐section of a mussel. A arrows indicate the general flow of water currents entering via the inhalant siphon and exiting via the suprabranchial cavity and exhalant siphon

To evaluate the changes in larval position in the mussels, the gills were divided into nine parts (Figure 1); from the gill demibranch to its point of contact with the suprabranchial cavity, the gill was divided into lower part (L), middle part (M), and upper part (U); it was also divided into three parts in the other direction, 3 being the farthest from the outlet, followed by 2 and 1. Moreover, the larvae’s position was accurately recorded and photographed (Canon, Mark II, Tokyo, Japan) by measuring the transverse length of the siphon of the mussel and the longitudinal length from the suprabranchial cavity to the gill demibranch. The developmental stages of *R. pseudosericeus* larvae were determined under a stereoscopic microscope (Nikon, SMZ‐10, Tokyo, Japan) using AxioVision LE program (version 4.5, Carl Zeiss, Germany), as described by Kim, Kang, and Kim (2006).

### 2.5 Observation of the larvae’s minute tubercles

The developmental dynamics of the minute tubercles at each larval developmental stage was determined by scanning electron microscopy (*SEM*), and the height of the minute tubercles was measured. For the *SEM* analysis, three specimens at each stage of larval development were fixed for 24 hr under cacodylate‐buffered 2.5% glutaraldehyde, dehydrated in an ethanol graded series, and dried to a critical point with liquid CO_2_. The dried samples were sputter‐coated with gold and then examined under an *SEM* (Supra40VP, Carl Zeiss, Jena, Germany). For photographic documentation and assessment of the minute tubercles, a Carl Zeiss vision camera (LE REL. 4.4; Carl Zeiss) was used for *SEM*.

**FIGURE 2:**
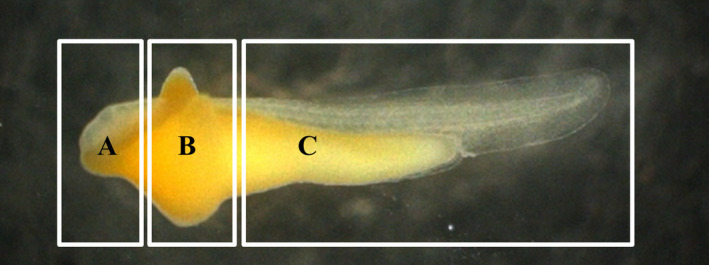
Diagram showing regions of the skin surface of larvae distributed the minute tubercles. (a) The eyes and head regions; (b) wing‐like projection; (c) posterior region of yolk sac

To facilitate the description of the distributional patterns, the surface of the larvae was divided into the following three regions (Figure 2) according to Kim et al. (2008): (1) anterior yolk sac projection covering the eyes and head (hereinafter referred to as EHR), (2) surface of the wing‐like projection composed of a pair of dorsal and one ventral yolk sac (hereinafter referred to as WLP), and (3) posterior regions of yolk sac and most parts of the body including the caudal fin‐fold region (hereinafter referred to as PR). During the 30 days of experiment, no dead mussels were found, and the minute tubercle height was measured at each larval developmental stage. Thirty‐minute tubercles per region were measured from three regions per larva removed from the tank.

### 2.6 Rhodeus pseudosericeus utilization of host mussel

Host use by *R. pseudosericeus* was determined by recording the position of larvae within the four gill demibranchs and the number and frequency of larvae. To compare the size of mussels with and without larvae, mussel shell length was measured to the nearest 0.01 mm.

### 2.7 Statistical analyses

Statistical analyses were conducted using SYSTAT software (Systat version 18.0, SPSS Inc.,, Chicago, IL, USA). A two‐sample *t* test was performed to compare the size of mussels with and without larvae. Kruskal–Wallis H test was used to test the difference in the number and frequency of larvae among different gill parts related to mussel size. Statistical significance was considered when *p* was < .05.

## 3 RESULTS

### 3.1 General features of the minute tubercles on R. pseudosericeus larvae

**FIGURE 3:**
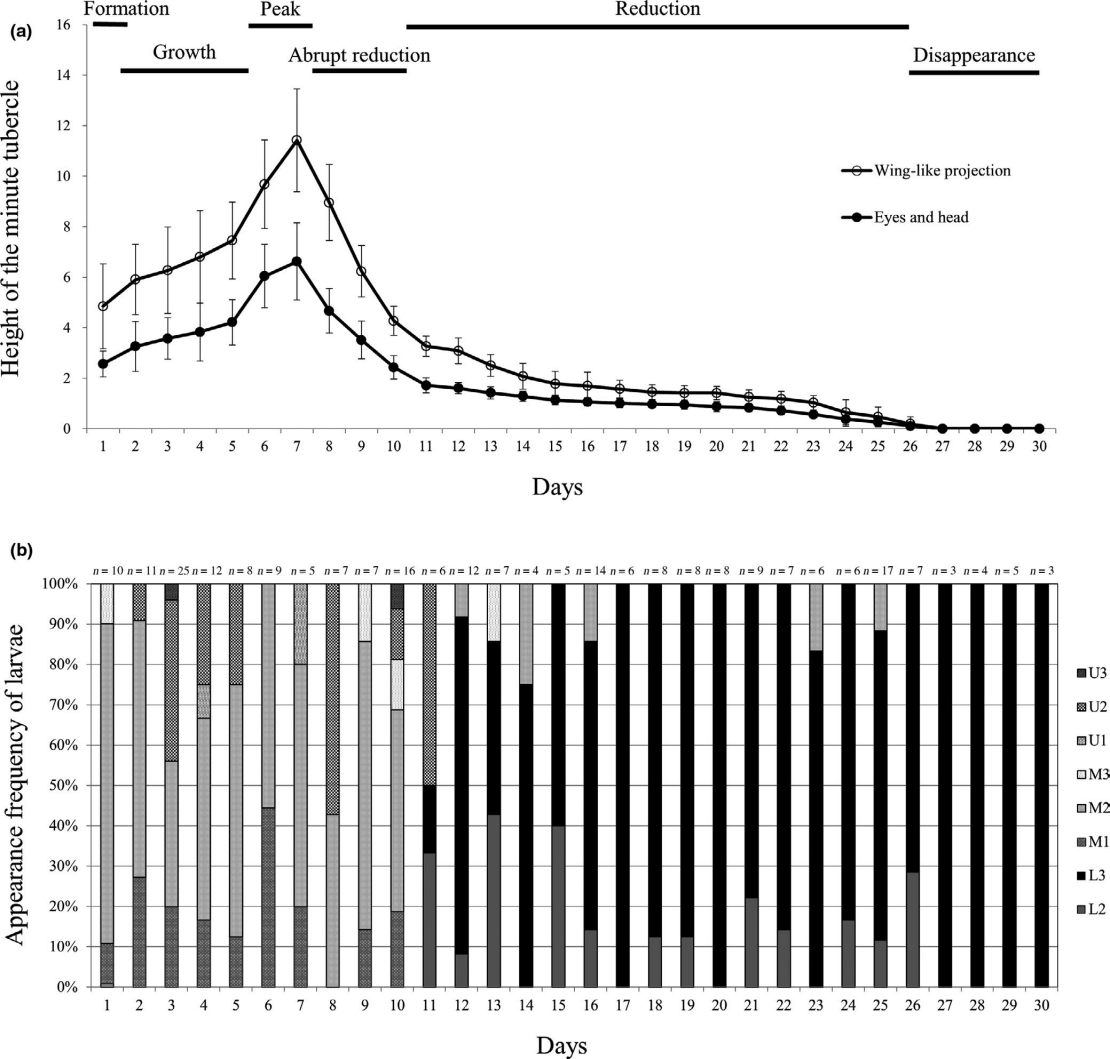
Mean (± *SD*) height of minute tubercle on the surface of two regions. (a) Eyes and head and wing‐like projection regions of *Rhodeus pseudosericeus* larvae; (b) the appearance frequency of *R. pseudosericeus* larvae in eight parts of gill demibranch inside the mussel. The color bars indicate the position of U1, U2, U3, M1, M2, M3, L2, and L3, respectively

**FIGURE 4:**
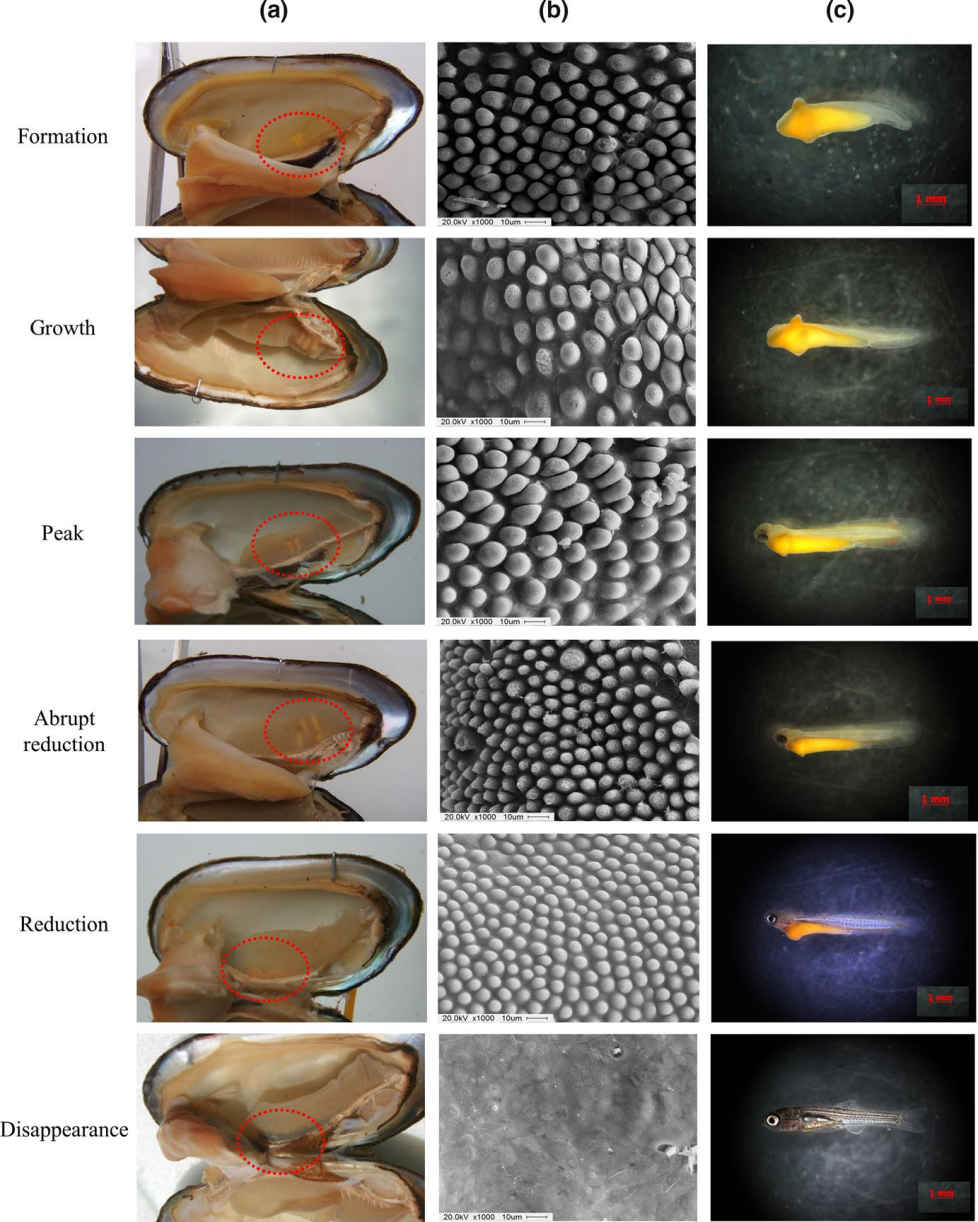
Diagram showing the position inside the mussels (a), development of minute tubercle on skin surface (b), and the morphological characteristics (c) of *Rhodeus pseudosericeus* larvae following the larval development stage. The dotted circle line indicates the position of larvae inside the mussels

The larvae’s minute tubercles were observed in the following three sites: EHR, WLP, and PR; the different heights of the tubercles in each region are shown in Table 1 and Figures 3, 4. The shape and degree of development of the minute tubercles clearly differed among regions. Two types of tubercles were found: hemispheric and vestigial shaped. The minute tubercles on the surface of EHR and WLP were hemispheric, whereas those on the PR were shrunken, flattened, and vestigial shaped. The tubercle distribution and development changed with larval growth and development.

**TABLE 1:** Mean (± *SD*) height of minute tubercles on the surface of two regions and characteristics of *Rhodeus pseudosericeus* larvae

Days after hatching	Developmental stage	Height of minute tubercles (µm)		Characteristics of larvae
		Eyes and head	Wing‐like projection	
1	Formation	2.57 ± 0.51	4.85 ± 1.68	No movement of larvae yolk sac begins to develop in the dorsal and ventral regions
2	Growth	3.26 ± 0.99	5.91 ± 1.38	Larvae start to move
3		3.57 ± 0.82	6.27 ± 1.71	Head slightly develops
4		3.83 ± 1.15	6.80 ± 1.83	
5		4.22 ± 0.90	7.45 ± 1.52	
6	Peak	6.05 ± 1.26	9.68 ± 1.76	Eyes form, heart beats
7		6.62 ± 1.53	11.42 ± 2.04	Red blood circulation is observed dorsal and ventral yolk sac shrink
8	Abrupt reduction	4.67 ± 0.88	8.95 ± 1.51	Minute tubercle contract and reduce
9		3.51 ± 0.75	6.24 ± 1.02	Caudal fin begin to develop
10		2.43 ± 0.46	4.28 ± 0.58	Lens completely develop
11	Reduction	1.72 ± 0.30	3.27 ± 0.40	Larvae in the suprabranchial cavity minute tubercle completely reduce
12		1.61 ± 0.23	3.09 ± 0.51	Pectoral and caudal fin develop
13		1.42 ± 0.24	2.51 ± 0.43	Air bladder divide into two rooms
14		1.28 ± 0.20	2.08 ± 0.51	
15		1.13 ± 0.19	1.78 ± 0.49	
16		1.07 ± 0.17	1.70 ± 0.54	
17		1.01 ± 0.19	1.57 ± 0.35	
18		0.98 ± 0.16	1.45 ± 0.28	
19		0.95 ± 0.17	1.41 ± 0.29	
20		0.87 ± 0.20	1.41 ± 0.26	
21		0.84 ± 0.16	1.25 ± 0.29	
22		0.72 ± 0.16	1.19 ± 0.29	
23		0.56 ± 0.13	1.03 ± 0.28	
24		0.38 ± 0.29	0.65 ± 0.50	Free‐floating individuals appear
25		0.26 ± 0.20	0.47 ± 0.38	
26		0.11 ± 0.16	0.19 ± 0.29	
27	Disappearance	–	–	All kind of fins completely develop
28		–	–	Anus open and
29		–	–	Yolk sac completely absorb
30		–	–	Larvae become free swimmers

The hemispheric minute tubercles on the EHR and WLP were observed immediately after hatching. Their height gradually increased from day 1 after hatching, with the highest values recorded on the WLP on day 7 [11.4 ± 2.0 (6.9–17.1) µm]. The height of the tubercles rapidly decreased by approximately 60% from days 8 to 10. The height of the tubercles continuously decreased from day 11, and on day 24, unidentifiable small protuberances were observed only around the eyes. No tubercles were observed on the epidermis of the larvae from day 27. The minute tubercles were mostly hemispheric but slightly inclined toward the posterior region; they were also denser and higher on the WLP than on the EHR. The minute tubercles on the posterior region were first observed on day 6, and they remained vestigial until day 11, when they shrunk or flattened.

### 3.2 Larval migration inside the mussels

The changes in larval position in the mussels in each larval developmental stage are shown in Table 1 and Figure 3. The spawning ovipositor of *R. pseudosericeus* entered the gill demibranch (U and M parts) or suprabranchial cavity (L part) of the mussel through the exhalant siphon (Figure 1), and the position of the larvae varied according to the developmental stage. Changes in the position of the larvae per developmental stage were different before and after day 11, when larval migration from the gill demibranch to the suprabranchial cavity was first observed. Until day 10, the larvae were found in the interlamellar space of the demibranch (U and M parts, prevalence 100%, *n* = 110); from day 11, the larvae were more common in the suprabranchial cavity (L part; prevalence 92.4%, *n* = 134) than in the gill demibranch (U and M parts; 7.6%, *n* = 11). The larvae in the L part were appeared to develop faster than those found in the M and U parts at the same time. All the larvae found in the gill demibranchs and suprabranchial cavities had their heads facing the direction opposite to the exhalant and inhalant siphons.

### 3.3 Relationship between the height and position of the minute tubercles and the morphological and physiological characteristics of the larvae

The position of larvae inside the mussels and the larvae’s external morphological and physiological characteristics were closely related as larval development progressed (Table 1, Figures 3 and 4). The changes in height of the minute tubercles were divided into the following six stages: formation, growth, peak, abrupt reduction, reduction, and disappearance.

#### 3.3.1 Formation stage (from hatching to day 1 after hatching)

During this stage, the EHR and WLP of the larvae were already covered with hemispheric minute tubercles. On day 1, the heights of the tubercles on the EHR and WLP were 2.6 ± 0.5 µm (1.8–4.1) and 4.8 ± 1.7 µm (2.3–8.9), respectively; that is, tubercles on the WLP were larger than those on the EHR. Tubercles on the PR of the larvae were not observed at this stage.

No larval migrations were detected immediately after hatching, but a pair of WLP, which were small and started to develop on the dorsal and ventral regions, were identified. The fin‐fold of the caudal region was very small at this stage. Moreover, all larvae were found in the M part of the mussels.

#### 3.3.2 Growth stage (days 2 to 5 after hatching)

At this stage, the minute tubercles developed very rapidly, reaching approximately twice their size in the previous stage, and they were abundant on the EHR and the WLP. Their heights on the EHR and WLP were 3.3 ± 1.0 (1.9–5.6) and 5.9 ± 1.4 (3.2–8.8) µm on day 2 and 4.2 ± 0.9 (2.7–7.1) and 7.5 ± 1.5 (4.5–12.0) µm on day 5, respectively. Vestigial minute tubercles began to appear in the posterior region, but were still very small.

On day 4, the larvae’s head developed slightly anterior to the egg yolk and the tubercles on the dorsal and ventral regions developed considerably. Between days 2 and 5, larvae were found only in the U and M parts of the mussels.

#### 3.3.3 Peak stage (days 6 to 7 after hatching)

The tubercles’ height was the highest at this stage (reaching approximately thrice their size in the formation stage), and their density on the EHR and WLP was very high. The heights of the tubercles on the EHR and WLP were 6.0 ± 1.3 (4.0–9.5) and 9.7 ± 1.8 (6.0–13.6) µm on day 6 and 6.6 ± 1.5 (4.2–11.0) and 11.4 ± 2.0 (6.9–17.1) µm on day 7—when they reached their peak heights—respectively. Vestigial minute tubercles on the PR were first observed during this stage, but they were still very small and flat.

The larvae began to form eyes, and their heartbeat could be observed under their heads. Red blood circulation could be observed in front of the yolk, and the epidermis on the dorsal side began to shrink slightly, with the yolk lengthening backwards. No larval migration was observed, and the larvae were only found in the U and M parts of the mussels.

#### 3.3.4 Abrupt reduction stage (days 8 to 10 after hatching)

At this stage, the minute tubercles on the EHR and WLP became considerably smaller and shorter than those in the previous stage. On day 8, the height of the tubercles on the EHR and WLP decreased to 4.7 ± 0.9 (2.8–7.0) and 9.0 ± 1.5 (5.2–12.9) µm, respectively; on day 10, it rapidly decreased to 2.4 ± 0.5 (1.7–3.8) and 4.3 ± 0.6 (3.1–5.7) µm, respectively, reaching a height similar to that of the formation stage.

At this stage, the development of the lens in the larvae’s eyes was completed, their heart components were clearly differentiated, and the caudal fin began to develop. The tubercles in the dorsal region were significantly contracted and shortened. The larvae remained in the demibranchs, and no migration was observed in the suprabranchial cavity. The larvae were only found in the U and M parts of the mussels.

#### 3.3.5 Reduction stage (days 11 to 26 after hatching)

At this stage, the tubercles in all sites were smaller than those in the previous stage. On day 11, the heights of tubercles on the EHR and WLP were 1.7 ± 0.3 (1.2–2.6) and 3.3 ± 0.4 (2.2–4.4) µm, respectively. On day 24, some larvae without minute tubercles were found. On day 26, almost all minute tubercles had disappeared, and only traces of them were left.

The pectoral and caudal fins of the larvae developed at this stage, and their eyes became clear and silver brown. Their heads, with complete upper and lower jaws, developed considerably. Their color darkened as the melanin pigment expanded, and their air bladders completely developed, with two parts and a slightly larger front. The tubercles on the anterior side completely reduced, followed by the reduction of those on the dorsal side; some parts of the yolk remained. Most of the larvae were found in the suprabranchial cavity. From day 24, free‐swimming individuals were found in the experimental tanks; the larvae that had remained inside the mussels also swam freely when removed from the mussels. A total of 2.3%, 6.2%, and 91.5% of the larvae were found in the U, M, and L parts of the mussels, respectively.

#### 3.3.6 Disappearance stage (day 27 to free‐swimming larvae)

At this stage, only parts of the minute tubercle were observed and only in some larvae. The pectoral, ventral, and caudal fins of the larvae were completely developed, mouth and anus were open, and yolk sac was completely absorbed. Larvae were found only in the L part of the mussels.

### 3.4 Host mussel utilization by R. pseudosericeus larvae

**FIGURE 5:**
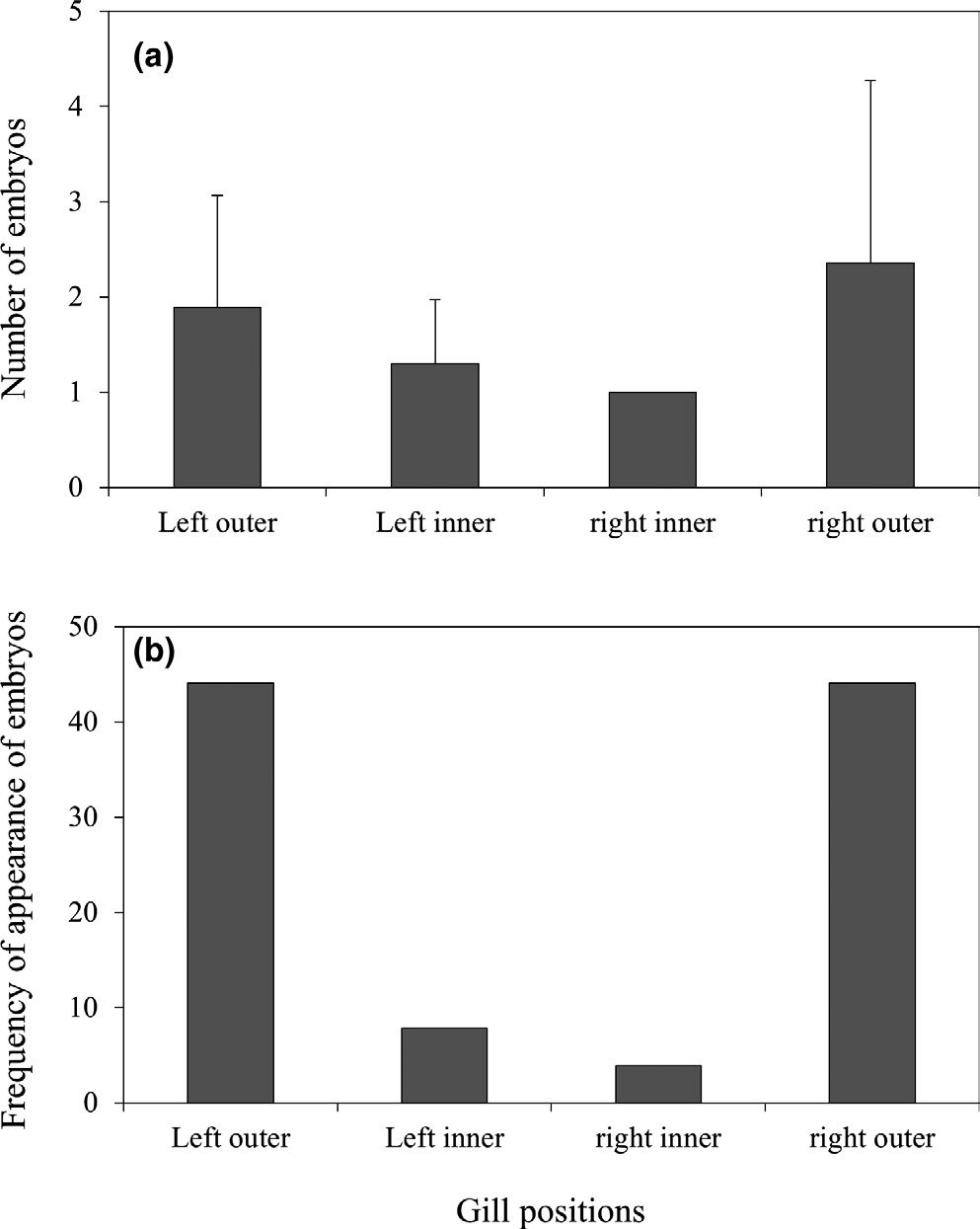
Mean (± *SD*) number (a) and frequency of appearance (b) of *Rhodeus pseudosericeus* larvae in the four demibranchs of gill positions inside the mussels

No significant difference in shell length was found between the mussels that had larvae (53.66 ± 6.65 mm; range, 38.42–68.82; *n* = 85) and those that did not (54.05 ± 7.20 mm; 40.10–69.96; *n* = 65; two‐sample *t* test; *t*_148_ = 0.341, *p* = .734). The number of larvae inside mussels was 3.01 ± 2.27 (range, 1–13; *n* = 127). The number of larvae in the left outer, left inner, right inner, and right outer gills of the mussels was 1.89 ± 1.17 (1–5; *n* = 56), 1.30 ± 0.67 (1–3; *n* = 10), 1.00 ± 0 (1; *n* = 5), and 2.36 ± 1.92 (1–10; *n* = 56), respectively (Figure 5a); no significant difference among the four demibranchs was found (Kruskal–Wallis *H* test, *p = *.148; Figure 5a).

A total of 49, 30, 6, and 0 larvae were found in one, two, three, and four parts of the mussels’ gills. The frequency of appearance of larvae in the left outer, left inner, right inner, and right outer gills of the mussels was 44.09% (*n* = 56), 7.87% (*n* = 10), 3.94% (*n* = 5), and 44.09% (*n* = 56) among each of the four demibranchs (Figure 5b). The larvae were significantly more frequent in the two outer demibranchs than in the inner demibranchs (Kruskal–Wallis *H* test, *p* < .001; Figure 5b).

## 4 DISCUSSION

In this study, I investigated the relationship among three factors (larval developmental stage, minute tubercle height, and position of the larvae inside the host mussel) (Table 1, Figures 3 and 4), and obtained three major results. Firstly, the minute tubercles were concentrated on the EHR and WLP of *Rhodeus* bitterling larvae, and they were more developed in the latter region than in the former. Second, during the formation, growth, peak, and abrupt reduction stages, larval development occurred in the interlamellar space of the demibranchs (U and M parts); larval migration to the suprabranchial cavity (L part) occurred only during the reduction stage, when the minute tubercles shortened (Figures 4 and 5). Finally, when the larvae migrated to the suprabranchial cavity, morphological and physiological changes related to their locomotion ability were apparent; in fact, individuals that migrated to the suprabranchial cavity clearly developed faster than those that remained in the demibranchs.

Two types of minute tubercles of *R. pseudosericeus* larvae were found, namely, hemispheric and vestigial shaped. Among *Rhodeus* bitterlings, *R. atremius*, *R. suigensis*, *R. ocellatus*, and *R. o. smithi* have only hemispheric minute tubercles, whereas *R. uyekii* and *R. pseudosericeus* have both hemispheric and vestigial‐shaped tubercles (Suzuki et al., 1985; Suzuki & Hibiya, 1984a; 1984b; Suzuki & Jeon, 1988b). The minute tubercles are unique features in bitterlings, and as wing‐like projections exist in *Rhodeus* bitterlings but not in *Acheilognathus* and *Tanakia*, they were used as a taxonomic characteristic to differentiate Acheilognathinae genera (Kim, 1982; 1997). Suzuki and Hibiya (1984a), (1984b) proposed the existence of three types of yolk projections in bitterlings, and *Rhodeus* was considered to have type‐C projections; this was confirmed in *R. pseudosericeus* in the present study. Suzuki and Jeon (1987) reported that the type and morphology of these minute tubercles change over time and from species to species. In the present study, *R. pseudosericeus* larvae with two shapes of minute tubercles similar to those of *R. uyekii* larvae were found; however, *R. pseudosericeus* larvae had a high proportion of hemispheric tubercles only on the EHR and WLP, whereas *R. uyekii* larvae developed these tubercles almost throughout the PR (Suzuki et al., 1985). The two species are very similar not only in the shape of the wing‐like projection and egg yolk during the development and disappearance stages, but also in the morphology of the adult fish; however, there were differences in the developmental area and height of the epidermis (Kim et al., 2006). Moreover, *R. pseudosericeus* eggs are not sticky and are laid in the interlamellar space of the demibranchs, whereas those of *R. uyekii* are sticky and laid in egg masses in the suprabranchial cavity (Kim, Ko, Choi, & Park, 2015). The reason for these similarities and differences cannot be determined based on the results of this study. Thus, in‐depth studies on speciation based on ecological characteristics and specific factors are necessary (Arai, Jeon, & Ueda, 2001; Myar, 1969).

I observed that the hemispheric minute tubercles on the WLP were approximately twice as large as those on the EHR, and the direction of the minute tubercles was slightly inclined posteriorly, making it easy for them to fixate on the gills but difficult to be removed, similar to a harpoon. The WLP was the largest and most developed part of the entire larva, with the largest surface area. The minute tubercles on the WLP began to develop shortly after hatching and began to shrink during the abrupt reduction stage. The hatched larvae that entered through the mussels’ exhalant siphon settled on the demibranchs, growing in their interlamellar space; during this stage, the widest surface area of the larvae is the WLP (Song & Kwon, 1994). Mortality of bitterling larvae occurs by two main factors: premature ejection by the mussel and death in the mussel gill by asphyxiation or nutrient deficiency (Kawamura & Uehara, 2005; Kitamura, 2005; Smith et al., 2000). The minute tubercles are formed by large unicellular epidermal cells and are presumed to be polysaccharidal in nature; studies have shown that they perform an attachment function that enables their attachment to vegetation and submerged objects (Laale, 1980). The minute tubercles occur only in larvae with no swimming ability; when the fins (and consequently, the larvae’s swimming ability) start to develop, the minute tubercles are abruptly reduced (Table 1, Figures 3 and 4). The minute tubercles in *Acheilognathus* and *Tanakia* bitterlings, which do not have a wing‐like projection, develop most intensively in the foremost part of the head, and the form of yolk projection is scaly or hilly, different from that of *Rhodeus* bitterlings (Fukuhara, Nagata, & Maekawa, 1982; Park et al., 2008; Suzuki & Hibiya, 1985; Suzuki & Jeon, 1987, 1988a, 1988c, 1988d, 1989, 1990). The development of larger and sharper minute tubercles in *Acheilognathus* and *Tanakia* larvae compared with those of *Rhodeus* larvae (20–40 µm versus. 3–15 µm) is an adaptation strategy that also prevents premature ejection and allows larvae to tightly fit in the interlamellar space of the hosts’ demibranchs (Kitamura, 2006b; Suzuki & Hibiya, 1985). In the present study, I confirmed that the change in the height of the tubercles on the HER and WLP was considerably related to the position of larvae in the mussels and that it may have an important role in preventing premature ejection by mussels. Further research will be required to compare the migration of *Acheilognathus* and *Tanakia* larvae inside mussels to investigate the role of minute tubercle by their types.

Mussels have one exhalant and one inhalant siphon. The bitterlings insert their ovipositor into the mussels’ exhalant siphon and lay eggs in the suprabranchial cavity or interlamellar space of the demibranch (Wu, 1998). As the inhalant siphon is connected to the mantle cavity, when the mussels’ shell opens, the larvae will be exposed to the environment; therefore, bitterling spawning must occur in the exhalant siphon to increase larval survival (Tankersley & Dimock, 1993a). The interlamellar space of the demibranchs expands as the larvae grow and it becomes a limiting factor. The larvae that remained in the interlamellar space for more than 11 days after hatching were found to have a slower development than those that migrated to the suprabranchial cavity. By migrating to the suprabranchial cavity, which is larger than the interlamellar space, ventilation rates can be increased, thus increasing oxygen supply and space (Davenport & Woolmington, 1982; Mills & Reynolds, 2002). Song and Kwon (1994) reported that *A. yamtsutae* larvae return to the U part as they gain physical abilities during the developmental stages. *A. signifier* and *R. sericeus* larvae, in contrast, remain in the interlamellar space only during the initial developmental stages, and as their swimming ability increases, they migrate to the suprabranchial cavity, in the direction opposite to the exhalant siphon (Aldridge, 1997; Back & Song, 2005). *A. rhombeus* has been reported to initially remain in the suprabranchial cavity, and then migrate in the direction opposite to the exhalant siphon (Kim, Choi, & Park, 2018). The bitterlings’ eggs inside the gills may compete with glochidia for oxygen and space (Kitamura, 2005; Smith, Rippon, Douglas, & Jurajda, 2001). The migration of larvae from the interlamellar space of the demibranchs to the suprabranchial cavity may reduce intraspecific competition and lower larval mortality rate in the suprabranchial cavity by providing space for growth and increased oxygen supply (Kitamura, 2006b; Methling et al., 2018; Spence & Smith, 2013).

Several previous studies have reported that mussel gill structure and conditions such as size, water flow speed, and dissolved oxygen content vary among gill positions, sexes, and density of larvae (Aldridge, 1999; Kitamura, 2005; 2006a, 2006b, 1993b; Mills & Reynolds, 2002, 2003; Smith et al., 2004; Tankersley & Dimock, 1993a). No glochidia were found during the present study, and therefore, the sex of the mussels was unknown. However, *R. pseudosericeus* larvae were mainly found in the two outer demibranchs of the four gills. *U. d. sinuolauts* is known to brood glochidia only in the outer demibranchs, but as the spawning season is after May, no glochidia care was observed during this study. Aldridge (1997) and Mill and Reynolds (2003) reported that the bitterlings mainly use the inner demibranchs, which had more larvae than the outer demibranchs, because of the following four reasons: active choice, space availability, ovipositor accessibility, and ejection ability. Studies have reported that *A. rhombeus*, *A. cyanostigma*, and *R. o. kurumeus* eggs were found at a higher rate in inner demibranchs than in outer demibranchs, suggesting that to avoid competition for oxygen and space with glochidia of mussels, these species use the outer demibranchs as brood pouches (Kim et al., 2018; Kitamura, 2006b, 2006c). Tankersley and Dimock (1993a) proposed that the total flow in the gills during brooding would be approximately 16% and 4% of those in nongravid and nonmarsupial gills, respectively. Kitamura (2006c) reported that female bitterlings may have been more constrained in spawning inside the inner demibranchs irrespective of mussel sex during group spawning. Moreover, Mills and Reynolds (2003) reported that when mussel brood larvae, bitterlings spawn in inner demibranchs, but after the mussels release their larvae, the widened outer demibranchs can be used as spawning sites. Interestingly, when the spawning patterns of mussels in March and April (i.e., before mussels brood the larvae) and in May and June (after the brooding season) were analyzed, *R. pseudosericeus* was found to have higher spawning rates in outer demibranchs than in inner demibranchs (per. observation). For *A. signifier*, twice as many larvae were identified in the inner demibranchs without brooding pouches compared with the outer demibranchs with brooding pouches (Kim, Yang, Ko, & Park, 2014). I proposed that the tendency to use the outer demibranchs of mussels by bitterling may be explained by the following three reasons: The cavity of outer demibranchs are linked to the mussel interior, in which the embryos are blocked, thereby making it difficult for the embryos to leave the mussel (anatomic structure); it is possible that the amount of dissolved oxygen and the circulation rate of water are higher (dissolved oxygen); and the growth rate of embryos is faster (growth rate). However, further studies are necessary to elucidate the selectivity of bitterlings regarding gill position and whether it is related to gill structure or active selectivity of bitterlings (Tankersley & Dimock, 1993b).

Bitterlings have a unique early life history. The bitterlings’ eggs can be classified into four types: bulb‐like, pear‐shape, spindly, and ovoid; moreover, some eggs are sticky (Kim et al., 2006). They lay a small number of eggs, develop unique tissue structures called minute tubercles during the early stages of larval development, have a very fast hatching time, and are unique in laying eggs in mussels. However, this lineage/clade/taxon, of which 60 species are known worldwide, evolved due to various factors such as maturation type, development, spawning type, spawning position and larval migration in mussels, and host selection (Nelson, 2006; Smith et al., 2004). In conclusion, in the present study, by examining the development of minute tubercles, migration of larvae inside mussels, and physiological characteristics of the larvae, I show that minute tubercles are developed to prevent the premature ejection of larvae by their mussel hosts. Thus, this finding may enhance our understanding of the evolutional advantages of the development of the minute tubercles and migration of larvae inside mussel for a better survival. In this present study, however, the investigation was limited to the determination of the main factors causing growth or reduction of minute tubercles development and advantages of migration of larvae. Therefore, further physiological research will be required to determine the role of physiological factors.

## CONFLICT OF INTEREST

None declared.

## AUTHOR CONTRIBUTION

**Hyeong Su Kim:** Conceptualization (equal); Data curation (equal); Funding acquisition (equal); Investigation (equal); Methodology (equal); Resources (equal); Software (equal); Writing‐original draft (equal); Writing‐review & editing (equal). 

## Data Availability

Data will be available at Figshare (https://figshare.com/s/10cfe2fee4bc89aeb268).
